# Self-injury in schizophrenia as predisposing factor for otomycosis

**DOI:** 10.1016/j.mmcr.2018.05.002

**Published:** 2018-05-03

**Authors:** Merad Yassine, Adjmi-Hamoudi Haiet

**Affiliations:** a Department of Parasitology-Mycology, CHU Hassani Abdelkader, UDL, Sidi-Bel-Abbès, Algeria; b Department of Parasitology-Mycology, HCA, University of Medicine, Algiers, Algeria

**Keywords:** *Aspergillus flavus*, Ear-injury, Otomycosis, Schizophrenia, Self-injury

## Abstract

Ear self-mutilation have been reported in schizophrenia, Mechanical damages to the auditory canal, like cleaning ear with hard and unsterile objects are predisposing factors of otomycosis.

We present a case of repeated self-induced auricular trauma in schizophrenic patient. Aural swab were collected and examined by direct microscopy and culture, revealing *Aspergillus flavus*. A traumatized external ear canal skin can present favourable condition for fungal growth in the psychiatric population.

## 1 Introduction

Otomycosis is a fungal infection of external auditory canal. Factors predisposing to this infection canal range from existing diseases, such as seborrheic dermatitis, eczema, psoriasis, climatic conditions and trauma [1].

They are different types of deliberate self-mutilating behaviours like self-cutting, bite, burns or ulcerations. Sometimes, especially among psychotic inpatient eye, tongue, ear or genital self-mutilation have been reported [2], we believe to be the first to report otomycosis in schizophrenia; it seems that this benign condition is neglected in the psychiatric population.

## 2 Case

**Fig. 1:**
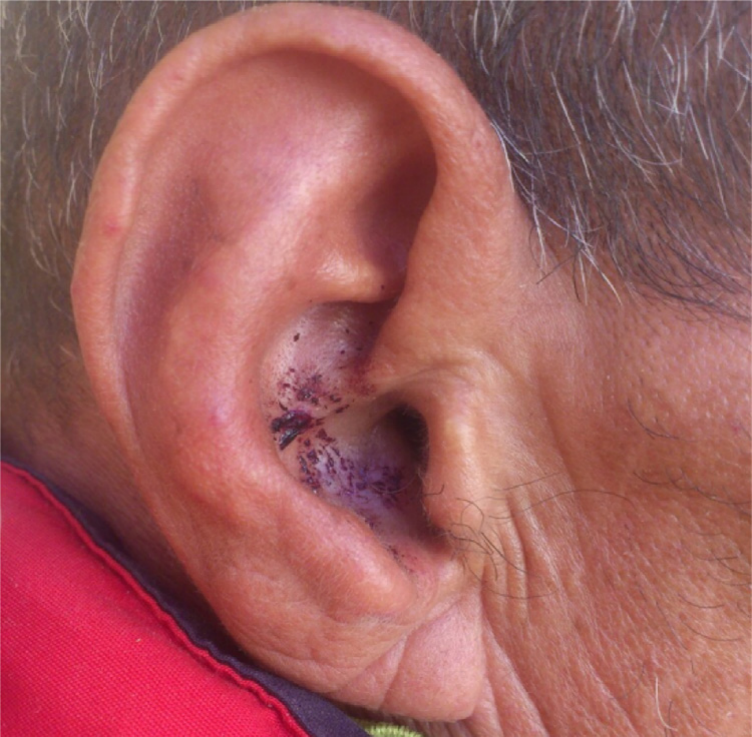
Ear self-injury in schizophrenic patient (day 2), before mycological examination.

A 56 year-old man with a 15 year history of schizophrenia, receiving inpatient care in a psychiatric unit, under continuous oral treatment with haloperidol (20 drops 3 times a day) presents multiple induced lesions of the auricle and the auditory canal (Fig. 1)

two weeks prior to examination the patient complain of itching and show skin lesions with erythema, scaling and red papules on the auricle, history of repeated ear self-injury was recorded, psychiatric staff noted that ear-self injuries were applied by fingers and wooden sticks from hospital garden, itching persists for more than two weeks ago. Moreover, there were no clinical signs of fungal infection on hands and nails.

The diagnosis of acute otitis externa was made in day 2, in the presence of itching on manipulation of the auricle and objective findings at otoscopy, such as the infection confined to the ear canal with greenish fuzzy growth and local area hyperhemic and bleeding.

Sterile cotton swab were collected under aseptic condition from external canal of the patient, the samples were inoculated on blood agar for routine bacterial culture and also on Saborauds Dextrose Agar (SDA) for fungal culture.

Direct microscopy by Lactophenol Cotton Blue (LPCB) wet amount preparation revealed septate acute angle dichotomous branching fungal hyphae.

**Fig. 2:**
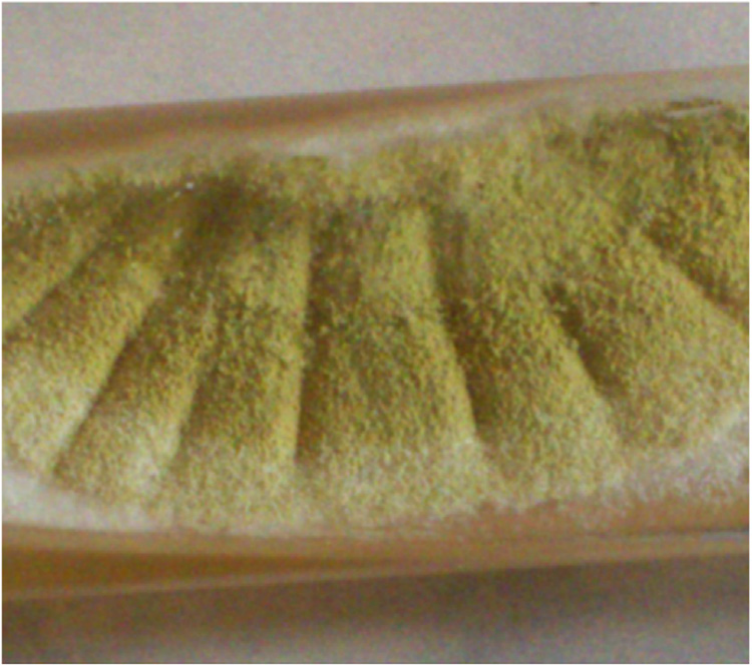
*Aspergillus flavus* culture macroscopy.

**Fig. 3:**
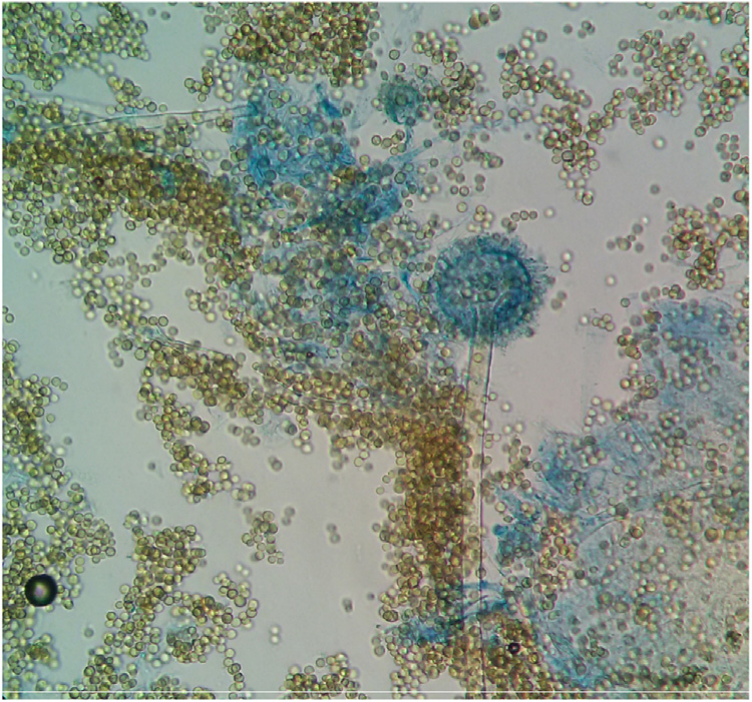
*Aspergillus flavus* microscopy X40: long conidiophore (400 µm) and rough, vesicle (40 µm), phialides arise circumferentially.

Routine bacterial culture was sterile, after 48 h of aerobic incubation. However on SDA after five days (day 7) of aerobic incubation, powdery masses of yellow-green colony developed (Fig. 2), the isolate was identified as *Aspergillus flavus, w*ith rough and colourless conidiophores and biseriates phialides (Fig. 3)

Present case was first managed by local toilet using dakin to remove the fungal debris from the ear canal. The patient was then started on clotrimazole 1% solution, ear drops were instilled into the affected ear three to four times a day during two weeks.

After one week (day 14) due to the agitation of the patient an oral treatment was initiated itraconazole 100 mg twice daily for 7 days. Instruction for the paramedical staff to monitor the patient so that he no longer handles the ear. The patient were reviewed after 1 week and the ear canal was observed under the microscope to assess the response to treatment at day 21.

Patient responded well to the treatment

Macroscopic colony morphology of Aspergillus flavus on SDA medium (day 10): surface is greenish-yellow with white border, texture is floccose

## 3 Discussion

Self-mutilations are considered as pathological after the age of three years [3] and ear self-mutilation have been reported in Schizophrenia [2], [4], on other hand, ear trauma is an established risk factor for otomycosis [5] and cleaning ear with unsterile objects (sticks, hair pin) or obsessive manipulation of the external ear canal with any hard objects such wooden sticks or metal wax picks have been reported as predisposing factors of otomycosis [6], [7]

Habit of cleaning the ear canal traumatized the skin and destroys cerumen protective barrier [8], [9], [10]

Trauma of the ear inoculates fungal debris, support the colonization of the auditory canal and deposition of fungal conidia in the wound causing fungal infection [11]

Damage to the auditory canal epithelium can lead to decreased excretion of the ceruminous and apocrine glands. These secretions maintain the protective properties of the keratin layers of skin by providing an acidic environment, which is bacteriostatic and fungistatic [12], [13], [14]; so damages will cause imbalance of the microbial flora of the auditory canal [15].

Aspergillus genus is the most common etiological agent of otomycosis, followed by Candida, many authors reported *Aspergillus flavus* as one of the most recovered species [1], [6], [11], [15], [16], this result is suggesting a possible relationship between ear self-injury and otomycosis.

Otomycosis is well known for its recurrence if proper treatment is not initiated [17]. Treatment such clotrimazole coupled with mechanical debridement are generally effective and the infection display a lower recurrence rate [18]

The case highlights situation of ear self-injury related to schizophrenia disorders and co-occurring otomycosis.
